# Case of a Foreign Body in the Air-Passages; Tracheotomy Performed for Its Removal

**Published:** 1852-03

**Authors:** David Johnston

**Affiliations:** Surgeon to the Royal Infirmary, Montrose


### RECORD OF MEDICAL SCIENCE.

### SURGERY.

*Case of a Foreign Body in the Air-Passages; Tracheotomy performed for its removal. Expulsion of the body twenty-eight days after the Operation, and recovery of the Patient.* By DAVID JOHNSTON, A. M., Surgeon to the Royal Infirmary, Montrose.—On Monday, May 5, 1851, John C——, aged fifteen, a clerk in a railway office, came from the country, a distance of nine miles, and applied to me for relief, relating the following circumstances:

Between seven and eight o'clock in the evening of the previous Thursday, May 1, while sitting with a sister, cracking and eating hazel-nuts, something occurred to cause a hearty fit of laughing, when he had a piece of nut-shell in his mouth, and the sudden inspiration caused the shell to disappear from the mouth and pass into the stomach, as he supposed. He was immediately seized with a paroxysm of suffocating cough, which lasted nearly an hour, and which left him in a state of extreme exhaustion. He slept during the night, although much annoyed with cough. Next day he felt pain about the throat, but no difficulty in swallowing. Towards evening his breathing became croupy and his voice husky. On Saturday, a surgeon who saw him administered an emetic, and passed a probang into the stomach, but found the œsophagus free from any obstruction.

When seen on Monday, the breathing was croupy and labored, and so loud and rough, that it could be heard outside the apartment in which the boy was; the voice was husky and could not be raised above a whisper; there was a constant hacking cough, accompanied by a little frothy expectoration; he complained of acute pain, increased by pressure, in the trachea, about an inch above the top of the sternum, and stated that the pain was more in the situation of the larynx during the two first days of his illness. He complained also, of a feeling of constriction in the chest, and of pain in the left side, referred to the neighborhood of the nipple. The skin was hot and dry, the pulse 108, and the respirations 24.

When stripped, the expansion of the thorax during inspiration was seen to be less complete than natural, and the ribs of the left side depressed. On applying the stethoscope over the trachea, a loud hoarse sound was heard, both during inspiration and expiration. The respiratory murmur was more audible over the upper part of the right lung than the left, and percussion also yielded a clearer sound over the right than the left side.

The diagnosis formed after this examination was, that the nut-shell had passed into the trachea, and that its outline being probably irregular and serrated, it had become entangled either in the trachea, at the spot where pain was felt, or in the left bronchus.

The serious nature of the case was explained to the boy and his friends, and it was recommended to them that he should remain in Montrose, where more speedy access could be had to assistance at any time, than in the country, at a distance of miles from any medical practitioner. To this proposal they assented.

May 6th.—Morning: Has had little sleep during the night, on account of cough, which is constant, but which does not occur in paroxysms; is unable to lie on either side; feels occasionally as if suffocation was impending, and obliged to start to the upright position; the countenance is anxious and pale, pulse 120. Evening: no better; was seen by a professional friend, who concurred with me in recommending tracheotomy, which was to be performed early next morning should no relief occur till then.

7th.—Eight A. M.: This morning, the seventh day after the occurrence of the accident, I opened the trachea, assisted by Messrs. Lawrence and Niddrie, surgeons. Fearing the probability of a considerable amount of blood finding its way into the air passage, it was deemed prudent to withhold chloroform. An incision of two inches in length was made exactly in the mesial line, between the cricoid cartilage and the sternum; a large turgid vein, which ran obliquely across the upper extremity of the wound along with the isthmus of the thyroid gland, was raised upon an aneurism needle, and pulled upwards out of the way of the knife, and the skin and other coverings of the tracheal tube divided. There was not the slightest hæmorrhage, and the trachea was immediately seized by a tenaculum, and opened to the extent of an inch and a half. After the lapse of a few minutes, an attempt was made to ascertain by a probe the situation of the shell. The irritation caused by this proceeding gave rise to such a paroxysm of spasmodic cough, that it was found impossible to persist in the exploration. The patient was allowed to rest a little, and another attempt made, but with the same result. With such force was the bloody mucus expelled from the wound, that the ceiling of the room was completely bespattered with it. At this stage of the proceeding, chloroform was administered, and with the happiest result. The trachea was now carefully examined with a probe, both above and below the wound; a pair of curved forceps, such as are used for extracting nasal polypi, with the blades closed, were introduced both into the right and left bronchus, but no foreign body could be detected. The little finger was passed upwards into the larynx, and its cavities examined; the tube, as far down as the bifurcation, was also hurriedly examined by the finger. The fauces and pharynx were also carefully explored, but still the object of search could not be found. All oozing of blood having ceased, the wound was now closed by stitches, covered with a pledget of lint soaked in tepid water, and the patient put to bed. Four P. M. No better: air and mucous escaping freely through the wound between the sutures; complains of sharp pain in the left side, in the neighborhood of the nipple; pulse 120; skin hot and dry; was again subject to the influence of chloroform, and the air-passages once more carefully examined, both with the finger and probe, with no more satisfactory result than before. The wound was more loosely closed than in the morning, and a dose of sulphate of magnesia with tartarized antimony was ordered to be taken in two hours. Nine P. M. Medicine has acted well; still complains of pain in the left side, and of a feeling of tightness over the whole chest; much teased with cough, which aggravates the pain; unable to expectorate on account of the escape of air through the wound; can only speak in a whisper; respiration abdominal. To have twelve leeches applied under the clavicles, and the following powder every three hours: submuriate of mercury, two grains; tartarized antimony, one eighth of a grain; compound ipecacuanha powder, two grains and a half.

8th.—Morning: has slept little; pain in side less severe; other symptoms much the same. The sutures were removed to allow free escape of mucus from the wound. Takes a little tea and toast. Evening: has been sick with the powders, but has not vomited.

9th.—Has passed a more comfortable night; can lie on either side to-day, but feels easier on the right; the chest expands very imperfectly, and the left side is flattened; the sounds are the same as before the operation. Pulse 100. The gums are beginning to get tender. He was ordered to take the powders only thrice daily, to have a dose of sulphate of magnesia immediately, and a blister under each clavicle.

10th.—He feels better, and his bowels have been freely opened; gums sore; copious discharges of mucus from the wound, but cannot expectorate by the mouth; still unable to speak, except in a whisper; pain in the side gone; has never felt the acute pricking pain in the throat since the operation; less stridulous and noisy. Pulse 96. Cough still very troublesome. To have a powder twice daily, and a tablespoonful, frequently, of the following mixture:—Syrup of squills, one ounce; ipecacuanha wine, two drachms; weak solution of morphia, one drachm and a half; camphor mixture, six ounces and a half.

11th.—Slept soundly five or six hours last night, and is beginning to relish food, which has been entirely farinaceous; still much annoyed with cough, and unable to lie on the left side, except for a few minutes; both blistered surfaces secreting pus freely, and wound granulating; the outward aspect of the thorax, as well as the sounds, continue without improvement. Pulse 80. Omit the powders, continue the expectorant mixture, and take a dose of castor-oil when necessary. To have beef-tea and an egg daily.

12th.—Same as yesterday: although the dulness and absence of respiratory murmur, as well as the flattened and immovable state of the left side of the thorax continued, the patient seemed to improve; his cough diminished, the breathing became more natural, the countenance lost, in a considerable degree, its anxious expression, the appetite returned, and he was able to pass nearly the whole of the day out of bed. This improvement went on progressively until the 17th, the day on which he ceased to breathe through the wound, and on which his voice recovered its natural tone; when all the amendment which had taken place was immediately dissipated, and the same train of inflammatory symptoms occurred which had proved so troublesome immediately after the operation. The same kind of treatment was again resorted to, and by the 31st the severity of the symptoms were again subdued. On that day a small abscess was opened on the left side of the neck, which discharged about a tablespoonful of pus.

June 4th.—This afternoon, between five and six o'clock, the patient was seized suddenly with a severe lacinating pain in the chest, behind the lower part of the sternum, accompanied by a paroxysm of cough, so severe that the by-standers expected instant dissolution. This lasted for twenty minutes, during which time I was sent for. By the time I arrived, however, all the symptoms of impending suffocation had vanished; and I had the satisfaction of finding the patient with the nut-shell, which had been such a source of danger and distress to him, in his hand instead of in his chest, where it had remained for a period of thirty-five days. It had been expelled by the mouth in coughing.

He now continued to improve steadily until the 13th of June, when he left Montrose for the country, with the wound almost cicatrized. When examined at this date, the cough and dyspnœa had altogether left him, but there still remained a degree of immobility of the parietes of the left side of the thorax, and the respiratory murmur was not so distinct on that as on the opposite side.

17th.—The young man was seen to-day, his chest examined, and no indication of disease found. The fragment of shell had an irregular, oblong form, and a rough, notched edge. It measured half an inch in length, and three-eighths of an inch across the widest part of its narrow diameter.

*Remarks.*—The history, as well as the physical examination of this case, left no room to doubt its nature, although the patient himself imagined that the shell had been swallowed: the immediate attack of suffocating cough, the continuance of dyspnœa, and other symptoms of interruption of the functions of the respiratory organs, after the œsophagus was found free from the presence of the shell, indicated its lodgment in some part of the air-passages. This opinion was confirmed by the aids afforded by the stethoscope and percussion. The uneasy feeling experienced by the boy about the middle of the trachea, seemed to indicate the probability of its being entangled in the mucous membrane in that situation, (and this had probably been the case during the first days of illness;) but the sounds produced by respiration and percussion, as well as the depressed and immovable state of the parietes of the left side of the thorax, pointed out with greater precision the left side of the bronchus, or some of its subdivisions, as the seat of fixture. All the symptoms led to the opinion that the shell was immovable; and its probable shape tended to strengthen this idea.

The amount of improvement which took place between the abatement of the inflammatory symptoms and the 17th of May, was calculated to lead to the belief that the offending body had been ejected unobserved along with the bloody mucus which was so forcibly expelled during the introduction of the probe before anæsthesia was induced; but the continuance of the unnatural position of the left lung, militated much against this opinion.

The blades of the forceps which were used to explore the bronchi, measured three inches and a half in length, and were introduced into each bronchus as far as the joint. It is possible that the shell, lying with its concavity closely applied to the mucus surface of the canal, and with its smooth convexity outwards, may have escaped detection with the forceps used as a probe; but the likelihood is that the body lay fixed deeper in the chest than it was possible to introduce them. It is almost impossible that it could have been undetected in those parts searched with the finger—viz., the cavity of the larynx and the whole length of the tracheal tube. It was obviously an oversight to close the wound, as the opening in the trachea afforded the patient the only available safeguard against suffocation, should the shell have suddenly become impacted in the rima. This error was, however, remedied on the morning after the operation.

This case serves to illustrate what is perhaps a novel application of chloroform. By a little delay, there is no doubt but the air-passages would have become enabled to endure with less resistance the irritation caused by the introduction of instruments, but never to allow of the accurate and prolonged examination, both by the probe and the finger, which was instituted. If the trachea of the patient is sufficiently large to admit the finger, and the wound sufficiently extensive to allow plenty of space for the carrying on of respiration after its introduction, the canal above the wound may be as carefully examined with the best and most delicate of all probes, as the mouth or fauces, without the slightest risk to the patient. The examination of the tube downwards must be performed hurriedly, and of course less satisfactorily. Notwithstanding the handkerchief held to the mouth and nostrils was repeatedly charged with chloroform, even to saturation, it was found impossible to induce insensibility until a sponge charged with the anæsthetic was applied to the wound.

Although the patient in this instance derived no benefit from the performance of tracheotomy, the risks he evidently encountered, and the termination of the case, clearly show that the operation was indicated. Some object to the performance of this operation under such circumstances as those related, except when the symptoms are imminent, from the fact that foreign bodies are often expectorated. But it is a fact equally well known, that in general, if they are allowed to remain for any length of time in such a situation, the constant irritation their presence gives rise to is the cause of serious mischief, which even their expulsion by expectoration fails to prevent from terminating in death, which, in such cases, is most frequently caused, not by suffocation from the foreign body getting entangled in the rima, or from any other sudden termination, but from phthisis, bronchitis, or some other tedious and destructive disease being induced by this cause.—*London Lancet.*

